# ﻿DNA barcoding reveals a taxonomic fraud: Note on validity of *Propomacrusmuramotoae* (Coleoptera, Scarabaeidae)

**DOI:** 10.3897/zookeys.1206.124932

**Published:** 2024-07-08

**Authors:** Seunghyun Lee, Seulmaro Hwang, Minhyeuk Lee, Jinbae Seung, Woong Choi, Ming Bai

**Affiliations:** 1 Key Laboratory of Animal Biodiversity Conservation and Integrated Pest Management, Institute of Zoology, Chinese Academy of Sciences, Beijing, 100101, China Institute of Zoology, Chinese Academy of Sciences Beijing China; 2 Department of Life Sciences, Natural History Museum, London, UK Natural History Museum London United Kingdom; 3 Department of Science Contents, Visang Education, Gwacheon, Republic of Korea Visang Education Gwacheon Republic of Korea; 4 National Institute of Agricultural Sciences, Wanju, Republic of Korea Seoul National University Seoul Republic of Korea; 5 Insect Biosystematics Laboratory, Department of Agricultural Biotechnology, Seoul National University, Seoul, Republic of Korea National Institute of Agricultural Sciences Wanju Republic of Korea; 6 305-403, Sechangnamsunhwan-ro, Namdong-gu, Incheon, Republic of Korea Unaffiliated Incheon Republic of Korea; 7 University of Chinese Academy of Sciences, Beijing, China University of Chinese Academy of Sciences Beijing China

**Keywords:** DNA barcoding, Euchirini, long-armed scarab, manipulated specimen, new synonymy

## Abstract

Until the early 2000s, the genus *Propomacrus* was known to comprise two species, occurring in the Eastern Mediterranean and Southeast China. The discovery of *Propomacrusmuramotoae* Fujioka in Tibet and subsequently in Bhutan and Nepal, might play a crucial role in bridging the geographical distribution gap of the Euchirini tribe between the Mediterranean and Central China, offering profound insights into its evolution and biogeography. However, all specimens, including the holotype specimen, were sourced from a single insect vendor, with no further specimens found or catalogued in museum collections thereafter. During our examination of a *P.muramotoae* specimen from a private collection in South Korea, we found its COI gene sequence to be identical to that of *P.bimucronatus* (Pallas) from Turkey, a species known for its wide distribution and genetic variability across regional populations. This overlap in genetic identity raised significant doubts, further compounded by our detection of deliberate modifications in essential diagnostic features during morphological examination. All three specimens we examined showed crude modifications, including staining and artificial grinding. Despite our inability to access the *P.muramotoae* type specimens for direct examination—a challenge we attempted to overcome through various means—it is evident that significant fraudulent tampering has occurred with the *P.muramotoae* specimens. Therefore, a new synonymy is proposed: *Propomacrusbimucronatus* Pallas, 1781 = *P.muramotoae* Fujioka, 2007 (**syn. nov.**). We also advocate for a straightforward verification of the type specimen through molecular analysis of the COI barcode region and morphological re-examination under a microscope for those who have access to the type specimens.

## ﻿Introduction

The beetle tribe Euchirini is characterized by their large size and notably elongated forelegs in males (Young 1989). These species are distributed widely across the Mediterranean, Indo-Asian Continental, and Southwest Pacific Insular regions (Young 1989). The tribe encompasses three genera: *Cheirotonus* Hope, 1841; *Euchirus* Burmeister & Schaum, 1840; and *Propomacrus* Newman, 1837. While *Euchirus* are confined to Southeast Asia and *Cheirotonus* spans Southeast Asia to the Indo-Himalayan region, *Propomacrus* exhibits the broadest yet distinctly disjunct distribution (Young 1989). *Propomacrusbimucronatus* (Pallas 1781), originally described from Turkey, has been recorded across a range of countries including Macedonia, Bulgaria, Greece, Turkey, Syria, Lebanon, Israel, Iran and Iraq (Young 1989; Muramoto 2012; Bezděk 2016; Ibrahim and Fayq 2022). *Propomacrusdavidii* Deyrolle, 1874, was described from central China, with its distribution is still restricted to that region. Alexis and Makris (2002) described *Propomacruscypriacus*, distinguishing it by male protibiae shape and ornamentation, which was later relegated to a subspecies based on mitochondrial DNA analyses, morphological reassessment and ecological data (Sfenthourakis et al. 2017).

A notable discovery within this genus was *Propomacrusmuramotoae* Fujioka, 2007, found in Tibet, with subsequent findings from Bhutan and Nepal (Muramoto 2012). This species would bridge the distribution gap between the Mediterranean and Central China, offering invaluable insights into the evolution and biogeography of *Propomacrus* and the tribe Euchirini. However, *P.muramotoae* was morphologically similar to *P.bimucronatus* and was described based on subtle morphological characters like the blunt lateral margin of the pronotum and a ventral groove on the abdomen (Fujioka 2007). Also, all specimens of *P.muramotoae*, including the type specimens, were obtained from Li Jingke, a beetle collector and seller known for altering locality labels (see Discussion). These circumstances raised significant doubts about the validity of this species, particularly given the absence of any subsequent findings. On the other hand, Sfenthourakis et al. (2017) provided extensive genetic resources for *P.bimucronatus* and a few DNA sequences of other Euchirini species, revealing broad genetic variation within *P.bimucronatus* and even the *P.b.cypriacus* population from Cyprus displayed multiple COI haplotypes. This suggests that confirming the validity of *P.muramotoae* could be straightforward with sequencing and comparison to existing public sequences.

This study aims to clarify the status of *P.muramotoae* by analyzing samples labeled as being collected from “Tibet” and “Nepal”.

## ﻿Material and methods

Three *P.muramotoae* specimens, deposited in the second author’s (S.H.) collection, one pair labeled as being collected from “Tibet” and one from “Nepal”, were used in this study. Specimens were examined with an Olympus SZ61 stereomicroscope and photographed with a DMC 5400 digital camera attached to a Leica Z16 APO motorized macroscope. Serial images were combined using Zerene Stacker.

**Figure 1.**
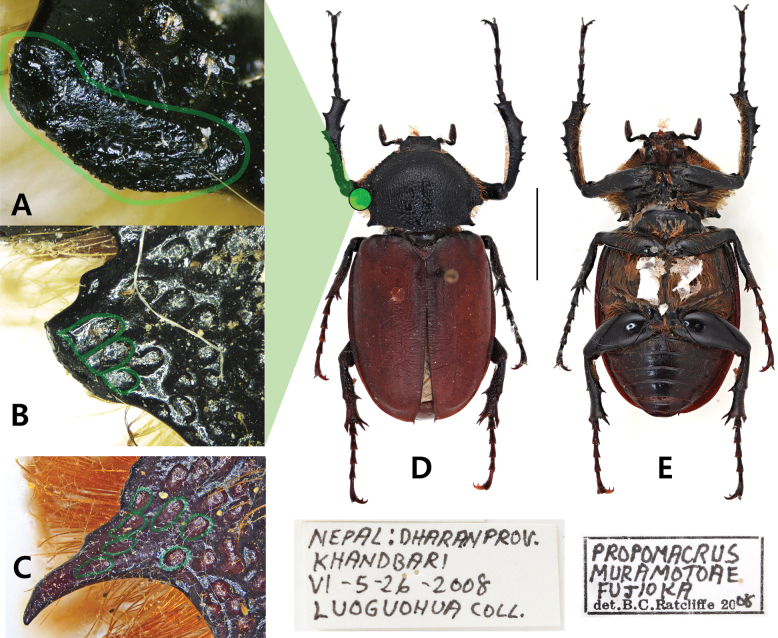
*Propomacrusmuramotoae* labeled as being collected from Nepal **A** lateral margin of pronotum, dorsolateral view. Light green lines highlight artificial grinding **B** lateral margin of pronotum, dorsal view. Light green lines highlight a punctation cut in the middle **C** lateral margin of pronotum of *P.bimucronatus***D** dorsal habitus **E** ventral habitus.

**Figure 2.**
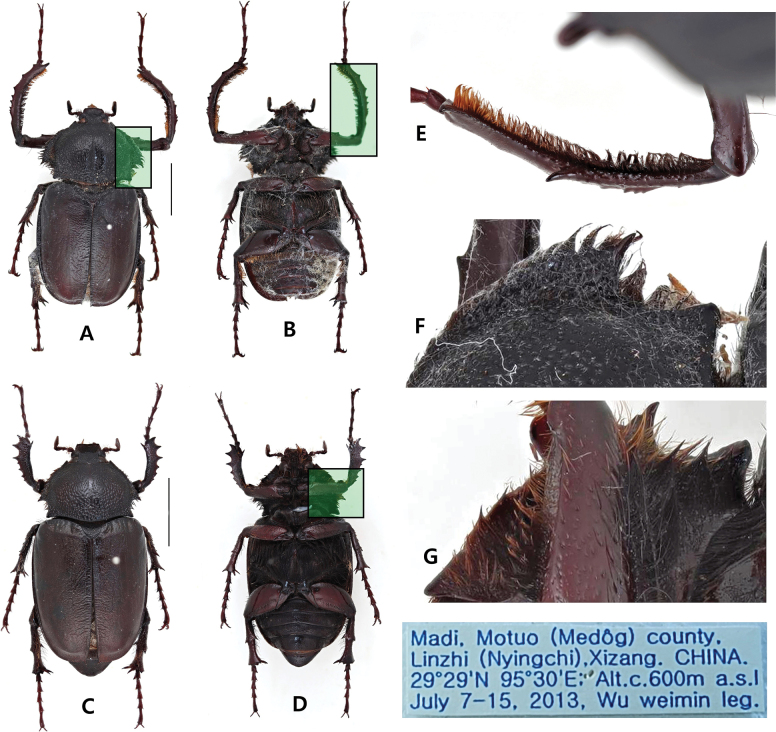
*Propomacrusmuramotoae* labeled as being collected from Tibet **A** dorsal habitus, male **B** ventral habitus, male **C** dorsal habitus, female **D** ventral habitus, female **E** forefemur of male with black stain **F** lateral margin of pronotum, dorsal view **G** lateral margin of pronotum, ventral view.

Genomic DNA from all three samples was extracted from both thoracic muscle and labial palpi of each specimen, using the DNeasy Blood & Tissue kit (Qiagen, Hilden, Germany), following the manufacturer’s protocol. The examined specimens were deposited in the private collection of the second author, and the collection labels’ details are provided in Figs 1, 2.

For compatibility with public sequences, we targeted the cytochrome oxidase subunit I (COI), previously utilized in a *Propomacrus* study (Sfenthourakis et al. 2017), to integrate our de novo data with the public data available on GenBank (https://www.ncbi.nlm.nih.gov/genbank/). As our samples were not in optimal condition, we initially retrieved all available COI sequences from GenBank and designed four new *Propomacrusbimucronatus*-specific primer sets. PCRs were performed using AccuPower® PCR PreMix (Bioneer, Daejeon, Korea) and sent to BIONICS Co., Ltd (Seoul, Korea) for sequencing. Public sequences used in this study, PCR primers, and PCR conditions are described in Suppl. material 1: tables S1–S3.

We utilized MAFFT ver. 7 online (Katoh et al. 2019) for multiple sequence alignment, and the final alignment was visualized in GENEIOUS (Kearse et al. 2012) to determine the position of each sequence. The amino acid translation option in MEGA X (Kumar et al. 2018) was used for the final sequence assessment. The phylogenetic analysis was conducted using the maximum likelihood method (ML) with IQ-TREE (Nguyen et al. 2015). Haplotype network analysis was performed using the TCS algorithm (Clement et al. 2000) implemented in PopART ver. 1.7 (Leigh and Bryant 2015). Sequences were categorized into six groups, representing each taxonomic unit (*Propomacrusmuramotoae*, *P.bimucronatusbimucronatus*, *P.bimucronatuscypriacus*, *P.davidis*, *Euchirusdupontianus* and *E.longimanus*).

## ﻿Results

### ﻿Morphological examination

The three specimens labeled as “*Propomacrusmuramotoae*” exhibited unusual morphological features (Figs 1, 2). Firstly, *P.muramotoae* labeled as being collected in “Nepal” exhibited notably blunt lateral pronotal processes as described in the original species description (Fig. 1D). The development of the elytral longitudinal costa was weak, and a ventral longitudinal groove was absent. However, microscopic analysis of the blunted areas on the lateral pronotal process indicated clear signs of artificial grinding. When observed from the side, the edges of the areas subjected to grinding were not smooth but were instead bluntly truncated throughout (Fig. 1A). The punctation on the plate was cut in the middle with a straight line across the area suspected of having been ground (Fig. 1B). The *P.muramotoae* specimens labeled as being collected from “Tibet” displayed a mottled black coloring across both sexes, appearing to be artificially dyed (Fig. 2A–G). Each specimen featured sharply developed lateral pronotal processes (Fig. 2F), with the elytral longitudinal costa weakly developed and a ventral longitudinal groove absent (Fig. 2B, D). These specimens have the diagnostic characters of *P.bimucronatus*, with the exception of their coloration.

### ﻿Molecular analyses

**Figure 3.**
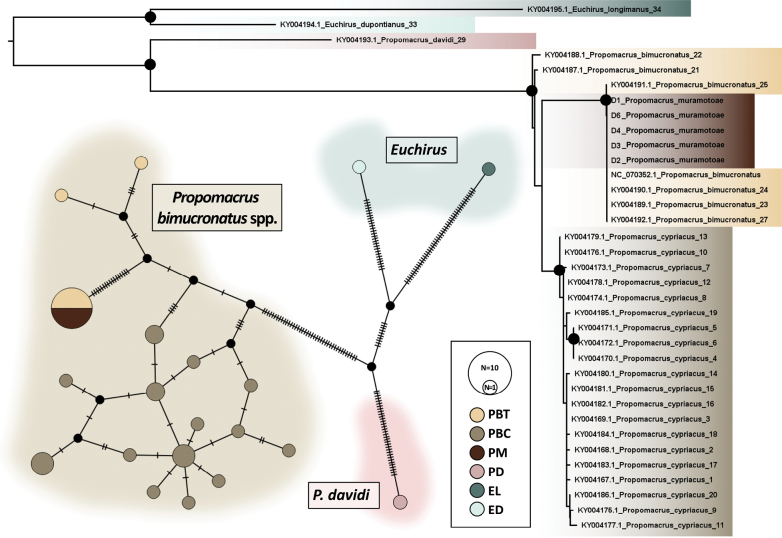
Genetic analyses using COI gene. **Lower left.** Haplotype network analyses. Abbreviations PM: *Propomacrusmuramotoae*, PBT: *P.bimucronatusbimucronatus*, PBC: *P.bimucronatuscypriacus*, PD: *P.davidis*, ED: *Euchirusdupontianus*, and EL: *E.longimanus*. **Right.** Phylogenetic relationships of *Propomacrus* resulting from IQtree. High Ultrafast bootstrap support values (≥90) are marked with black circles.

In the network analysis, we identified a total of 17 haplotypes within the *P.bimucronatus* species complex (*P.b.bimucronatus* + *P.b.cypriacus*). Within these, *P.b.cypriacus* exhibited notable diversity, presenting 14 distinct haplotypes. Conversely, only two haplotypes were observed in *P.b.bimucronatus*. Notably, a predominant haplotype, designated as haplotype A, was shared by the majority of individuals studied. This haplotype was particularly significant in our analysis of *P.muramotoae*; all five sequences examined were identical to haplotype A. In terms of haplogroups, *P.b.cypriacus* formed a distinct group, while the remaining sequences recovered polyphyletic (Fig. 3).

Furthermore, monophyly of *P.bimucronatus* species complex was recovered with strong Ultrafast Bootstrap Support (UBS = 100) within the maximum likelihood (ML) tree. In the phylogenetic tree, *P.muramotoae* was clearly nested within the *P.bimucronatus* clade. The clade, which included five *P.muramotoae* specimens was monophyletic with a branch length of zero and high support values (UBS = 99). Consistent with the network analysis, the *P.b.cypriacus* clade formed monophyletic groups with high supporting values (UBS = 90), reinforcing the results observed in the haplotype analysis (Fig. 3).

## ﻿Discussion

Our DNA analysis showed a variety of haplotypes of *P.b.cypriacus* among the extensive samples from the small island of Cyprus. Conversely, *P.b.bimucronatus*, with a distribution over a significantly larger area, has a disproportionately small number of sequences uploaded to GenBank relative to its range, with all specimens collected in Turkey. Therefore, widely distributed *P.b.bimucronatus* should exhibit higher genetic diversity than *P.b.cypriacus*, as a wider range correlates with greater genetic diversity in close congeners (Cole 2003; Leffler et al. 2012; Hague and Routman 2016). Furthermore, existing studies of the genus *Cheirotonus*, which is closely related to *Propomacrus* (Yu et al. 2023), demonstrate significant intraspecific variation in genetic diversity correlated with its species’ distribution patterns: *Cheirotonusgestroi* Pouillaude, 1913, which has a wide distribution, shows a broad range of genetic variation (Yang et al. 2020), in contrast to *Cheirotonusformosanus* Ohaus, 1913, which has a narrower distribution and exhibits lesser genetic variation (Huang et al. 2024). It is particularly unconvincing that individuals found with the labels “Nepal” and “Tibet” possess a COI haplotype identical to the most common haplotype identified in Turkey populations, raising suspicions about the uniformity of sequences between the individuals.

The morphological characteristics of the species are also notably ambiguous. The lateral pronotal process considered a distinctive feature of *P.muramotoae*, was only observed in one specimen, where it appeared to have been artificially modified. This modification is particularly prominent in a sharply cut punctuation along the lateral margin. The presence of the elytral longitudinal costa, a trait often found in *P.bimucronatus*, adds to the ambiguity, along with the absence of the abdominal longitudinal groove in all specimens examined. The remaining two specimens, labeled as from Tibet, were indistinguishable from *P.bimucronatus* in both DNA and morphological aspects and lacked any diagnostic features of *P.muramotoae* according to the original description.

It is essential to recognize that all specimens of *P.muramotoae* were exclusively provided by an insect dealer, Li Jingke (personal communications with the second author). Li Jingke has a well-documented reputation as a fraudster (Suppl. material 2: fig. S1) even though his fraudulent activities have rarely been formally reported (Han et al. 2017). Probably, the specimens were reared from larvae or obtained from common sources, such as bred population from Turkey, and subsequently altered to sell at high prices. Surprisingly, in several advertising emails from Li Jingke that we received, we found descriptions of specimen variations that appeared completely random, such as white lines on the elytral margin, wider elytra, and a bleached posterior half (Suppl. material 2: fig. S2). Specimens advertised as having these ‘unique features’ were sold at very high prices (Suppl. material 2: fig. S2). Such practices, though inconceivable within the scientific community, unfortunately do exist. Direct manipulation of specimens is rarely documented in entomology (Braby and Eastwood 2019). The morphological alterations by the fraudster were carelessly executed in this case, and fortunately, DNA barcode amplification was successful. However, it should be noted that such verification may not always be possible.

Based on genetic and morphological analysis, coupled with indirect data discussed above, we believe that the type of *P.muramotoae* is an altered specimen of *P.bimucronatus*. Therefore, we propose that *P.muramotoae* Fujioka, 2007, is a junior synonym of *P.bimucronatus* Pallas, 1781. A significant limitation of our study, however, is the absence of examination and genetic analysis of the type specimens. The type specimens of *P.muramotoae* are housed at the National Museum of Nature and Science in Tokyo, Japan, according to the original description. However, we were unable to find the types for our research; they were not deposited at the National Museum of Nature and Science and it is presumed they remain within the collection of the original describer. All authors tried to contact him in various ways but failed to access the type specimens. The lack of genetic divergence from *P.bimucronatus* and clear evidence of morphological manipulation strongly indicate that *P.muramotoae* represents a significant taxonomic deception. Our research indicates that verification of the type specimen is feasible and straightforward and we suggest those with access to the holotype conduct official taxonomic verification of *P.muramotoae*: simply amplify and do molecular analyses using the COI barcode region and examine external morphology under a microscope.

## ﻿Taxonomic account

### ﻿Tribe Euchirini Hope 1840

#### ﻿Genus Propomacrus

Taxon classificationAnimaliaColeopteraScarabaeidae

Newman, 1837

AB7A0D1E-B24C-5A18-8D4B-1D513B13CA5E

Porropus Laporte 1840: 113.
Protomacrus Hope 1841: 595.
Macropropus Agassiz 1846: 309.

##### Type species.

*Scarabaeusbimucronatus* Pallas, 1781: 13.

#### ﻿ Propomacrus bimucronatus bimucronatus

Taxon classificationAnimaliaColeopteraScarabaeidae

Pallas, 1781

1E6308E1-17C8-5A7E-B762-D21F3ED9C591

Scarabaeus bimucronatus Pallas, 1781: 13.
Propomacrus arbaces Newman, 1837: 256.
Propomacrus muramotoae Fujioka, 2007: 99. (syn. nov.)

##### Material examined.

**Turkey** • 1 male, 2 females; Mersin province 1500 m nr. Köseçobanli village dead in old pollarded oaks; 2017; Serder Göktepe leg.; BMNH{E} 2018-74; Natural History Museum London (NHM hereafter) • 1 male; Smyrna; NHM • 1 male; Asia Minor; 1910; G.a. Tellalian; NHM • 1 female; Asia Minor; NHM • 1 male, 2 females; Fry coll.; As Min Smyrne; 1905-100; NHM • 1 female; Besika Bay; G.C.C. Champion; 1927-409; NHM • 3 males, 1 female; Smyrne; 1906; Chinese Academy of Sciences • 2 males, 2 females; Hatay; Jun. 2007; private collection of Woong Choi. **Syria** • 2 females; Syria; 80.53; NHM • 1 female; Aleppo, Syria; G Lewis; 1915-38; NHM.

##### Additional material with falsified labels.

**Nepal** 1 female; Khandbari, Dharan Prov.; 5 Jun. 2008; LUOGUOHUA leg.; private collection of Seulmaro Hwang; **China** 1 male, 1 female; Madi (Medog) county, Linzhi (Nyingchi), Xizang; 29°29'N, 95°30'E; alt. c. 600 m; 7–15 Jul. 2014; Wu Weimin leg.; private collection of Seulmaro Hwang.

#### ﻿ Propomacrus bimucronatus cypriacus

Taxon classificationAnimaliaColeopteraScarabaeidae

Alexis & Makris, 2002

426EE80D-3A4F-57C5-B768-E9127E19AAE6

Propomacrus cypriacus Alexis & Makris, 2002: 103.

##### Material examined.

**Cyprus** • 1 male; Alethriko, Larnaca; 34°51.54'N, 33°29.38'E; 15. viii. 2006; Aristos Aristophanous leg.; BMNH{E}2015-88; NHM • 1 female Alethriko, Larnaca; 34°51.54'N, 33°29.38'E; 5 ix 2008; Aristos Aristophanous leg.; BMNH{E}2015-88; NHM.

## Supplementary Material

XML Treatment for Propomacrus

XML Treatment for Propomacrus bimucronatus bimucronatus

XML Treatment for Propomacrus bimucronatus cypriacus

## ﻿Additional information

### Author ORCIDs

Seunghyun Lee https://orcid.org/0000-0001-6318-4116

Minhyeuk Lee https://orcid.org/0000-0002-5667-9097

Jinbae Seung https://orcid.org/0000-0001-9115-1733

Ming Bai https://orcid.org/0000-0001-9197-5900

### Data availability

All of the data that support the findings of this study are available in the main text or Supplementary Information.

## Supplementary materials

Supplementary material 1 Public sequences, PCR primers, and PCR conditions Data type xlsx https://binary.pensoft.net/file/1087165This dataset is made available under the Open Database License (http://opendatacommons.org/licenses/odbl/1.0/). The Open Database License (ODbL) is a license agreement intended to allow users to freely share, modify, and use this Dataset while maintaining this same freedom for others, provided that the original source and author(s) are credited.Seunghyun Lee, Seulmaro Hwang, Minhyeuk Lee, Jinbae Seung, Woong Choi, Ming Bai

Supplementary material 2 Deceptive practices of Li Jingke and sales email from him Data type pdf https://binary.pensoft.net/file/1087166This dataset is made available under the Open Database License (http://opendatacommons.org/licenses/odbl/1.0/). The Open Database License (ODbL) is a license agreement intended to allow users to freely share, modify, and use this Dataset while maintaining this same freedom for others, provided that the original source and author(s) are credited.Seunghyun Lee, Seulmaro Hwang, Minhyeuk Lee, Jinbae Seung, Woong Choi, Ming Bai
