# Supplementary material for: ﻿DNA barcoding reveals a taxonomic fraud: Note on validity of Propomacrusmuramotoae (Coleoptera, Scarabaeidae)
Source: Zookeys. 2024 Jul 8;1206:181–90. doi: 10.3897/zookeys.1206.124932 (PMC11249845; doi:10.3897/zookeys.1206.124932)

Figures S1. Deceptive practices of Li Jingke

https://archive.insectnet.com/thread/945/beware-jingke-li-active-again
https://archive.insectnet.com/thread/817/papilio-machaon-ssp

« Prev 1 Next » Actions Search...

lucanidae25
Darwin

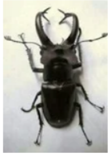

Posts: 1,030

Apr 20, 2011 at 12:11am Quote

Be very careful with these eds:

www.eurofauna.com/invertebrates/dried-insects

Jingke Li is back to his old trick again by using the materials that he gets from Laos, China and fakeing datas from Myanmar and Buthan in order to sell them.

Last Edit: Apr 20, 2011 at 12:13am by lucanidae25

---

bichos
Darwin

bichos Avatar

Posts: 1,085

Apr 21, 2011 at 8:34am Quote

> *Apr 21, 2011 at 4:42am* @thanos said:
>
> I have just received another email from Jingke (carabuslaos@yahoo.com.cn),with topic 'New Wholesale Beetles and Lepidoptera,parnassius specimens' ,from 'laos office' ,offering many different mixed lots with datas..,also a list of Carabidae and Lucanidae,and a book :
> 'By Li Jingke,2005,<The Carabinae of China,>' ....
>
> Is he a swindler ? I've never dealt with him or replied to his emails.
>
> Thanos

Yeah! he has not deactivated his activities at all judjing by the e-mails no.
Not a swindler as such but rather a dealer , packing is rudementary. and he has been the source of unreliable data, he does deliver however and it is very tempting
his forte would be carabidae from what I understand...

It seems therefore that a taste for collecting beetles is some indication of future success in life!--Charles Darwin

---

Adam Cotton
Moderator

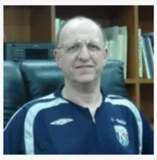

Posts: 3,496
Country: Thailand

Apr 21, 2011 at 4:46pm Quote

Unreliable data is a total understatement!!! He deliberately fakes data in order to sell cheap specimens for higher than normal prices. Try doing a search for Li Jingke in the old forum, and you'll find some examples there.

Just for an example he was selling $5 Papilio krishna charlesi (from Sichuan) for $30 each with data "Pongsaly, N. Laos". He also sold Sichuan P. bootes ($3 each in Sichuan, maybe) with the same data for $20 each. I have examined specimens and not only are they clearly from Sichuan, but the bootes were specifically selected (white spotted form only, black form would be too obvious to most collectors).

I know of many other similar fake specimens, and to be blunt, his actions are fraudulent and in any other business they would be acted upon by the police. Sadly in future such specimens with fake data will end up in museums, and researchers will not realise the data is fake.

Adam.

---

timoinsects
Nabokov

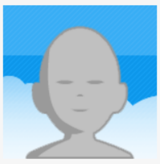

i collect beetles specimen

Posts: 991

Apr 25, 2011 at 1:50pm Quote

here i add more:

a tel calling came from a native dealer around a month ago, he asked me if i have mixed beetles (cheap price) for sale,he needed chinese beetles. i had no chinese mixed beetles actually. i didn't asked him why he was looking for chinese mixed beetles from me,then later another of my friend(also a local dealer) told me the recipe was that LJK bought from them at cheap wholesale price and was in big needed frequently. guess was it possible for one dealer collected himself in wild so had bulk insects from many different places with long distance each other? NO! this tells it has already been impossible to tell a exact correct data.

actually the specimen LJK got from severl different local dealers,and the insects originally came from different places or even provances,and mixed them together in a same alcohol container or so... this means nobody could tell their collecting data as a whole at all. a mixed insects came from different places at different date for one same data??NO!

that's just one of the aspects i know.

Last Edit: Apr 25, 2011 at 2:01pm by timoinsects

i collect tropical beetles(lucanidae,cetonidae,cerambycidae,prionidae,dynastidae,buprestidae,tenebrionidae etc.),and one-beetle groups like grasshoppers,phasmids,cokroachs.etc.

---

wollastoni
Moderator

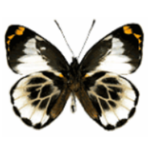

Posts: 2,390
Country: France

Apr 26, 2011 at 10:43am Quote

A serious seller in China with accurate data would earn a lot of money... too bad, no Chinese man has understood it for the moment.

Another example, Delias lattivita formasana lives only in Taiwan. It is offered from Sichuan by a Chinese seller on Ebay. I kindly inform him his identification was false and that he may receive complaints from potential bidders.

--> He answered me he was 100% sure formosana fly in Sichuan ...

The TOP 100 INSECT AUCTIONS : collector-secret.com/top-insect-auctions/
Delias of the World : delias-butterflies.com
My blog about insect photography : macrophoto-insectes.blogspot.fr/

Adam Cotton
Moderator

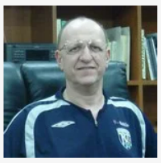

Posts: 3,496
Country: Thailand

Mar 27, 2011 at 8:39am Quote

The Korean subspecies is hippocrates. Bryk proposed a new name for Korean machaon, kaolimachaon Bryk 1946, but it's not different from hippocrates. There is also a name koreae Eller 1939, but this is a nomen nudum as there was no description in the publication, only a name, even though there are 'type' specimens in the NHM, London.

Beware that there are specimens on the market with fake data supposedly from North Korea which have originated from Li Jingke. He sold them on to various dealers around the world. I got some from Roman Yakovlev and only realised after receiving the specimens that the data was probably wrong, and typical Li Jingke style:
Mt. Huisaek Peak, Ryanggang province, North Korea. June1-16, 2008.
There was a machaon in with these specimens that doesn't look like Korean machaon at all!

Adam.

---

wollastoni
Moderator

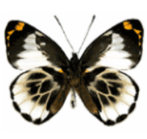

Posts: 2,390
Country: France

Mar 27, 2011 at 1:06pm Quote

Li Jingke is a burden...

The TOP 100 INSECT AUCTIONS : collector-secret.com/top-insect-auctions/
Delias of the World : delias-butterflies.com
My blog about insect photography : macrophoto-insectes.blogspot.fr/

Figures S2. *P. muramotoae* sales email from Li Jingke

Email 1: 21. Jan. 2012

from Laos office:
Data: Thrumshingla National Park, Mongar city, Buthan. June20-27, 2010
Propomacrus sp(A2 quality): 1500$/only 1male
postage$40, total is 1540$
this specimens is different with other world recored Propomacrus, its body is very wide, the Elytra of two side are white, you can see from the photo.

==

Data: Ma-ma-luo, zayu county, Tibet. June1-26,2010,
Propomacrus muramotoae

No.	mm	US$/1 ex
male		
1,A1	39	600
2,A1	29	300
3,A1	34	500
4,A1	29	300
5,A2	30	200
6,A2	34	300
female		
7,A1	35	300
8,A1	31	200
9,A1	33	250
10,A1	33	250
11,A2	35	150

Email 2: 4. Jun. 2009

Data: Khandbari, Dharan province, Nepal.june12-30,2008
Luoguohua Leg.
Propomacrus muramotoae ssp(Red Elytra) 7male=3000$

yours
Li Jingke

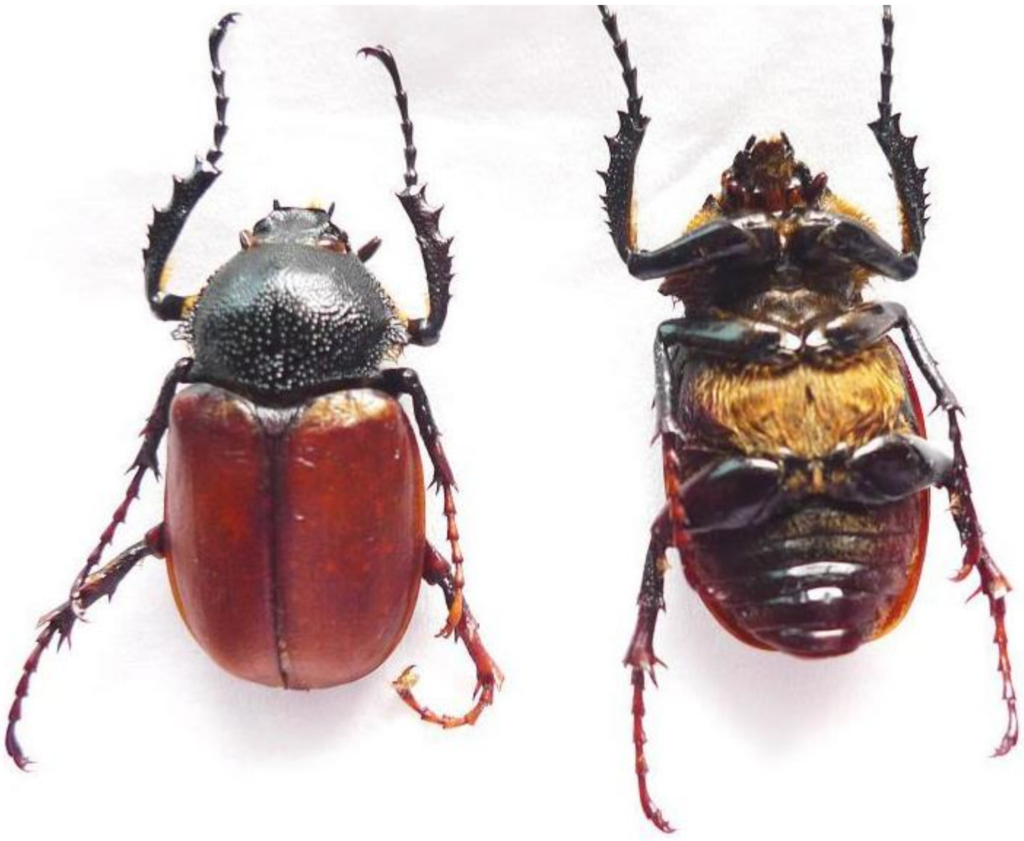

Supplement: Supplementary material 2 — Deceptive practices of Li Jingke and sales email from him [file zookeys-1206-181_article-124932__-s002.pdf]
